# Supplementary material for: Are carbon emissions trading and green financial instruments synergistic? -Comprehensive quantitative research based on content analysis
Source: PLoS One. 2024 Mar 7;19(3):e0298601. doi: 10.1371/journal.pone.0298601 (PMC10919601; doi:10.1371/journal.pone.0298601)
Supplement: S1 Data — (ZIP) [file pone.0298601.s001.zip › Data/地级市Z_F工作报告.zip!/╣≤╓▌/┴∙┼╠╦«╩╨/└┤╫╘╣1⁄2╓┌║┼í╛┬φ┐╦╩2╛▌═°í┐_2006.docx]

各位代表：

　　根据《中共六盘水市委关于制定六盘水市国民经济和社会发展第十一个五年规划的建议》，市人民政府制定了《六盘水市国民经济和社会发展第十一个五年规划纲要（草案）》。现在，我代表市人民政府向大会报告，请各位代表连同《纲要（草案）》一并审议，并请各位政协委员和列席的同志提出意见。

“十五”时期国民经济和社会发展回顾

　　过去五年，在省委、省政府和市委的领导下，在市人大、市政协的监督、支持下，我们坚持以邓小平理论和“三个代表”重要思想为指导，全面贯彻科学发展观和构建社会主义和谐社会的重大战略思想，牢牢把握加快发展这个富民兴市的第一要务，紧紧抓住西部大开发和新阶段扶贫开发的机遇，团结和依靠全市各族人民，聚精会神搞建设，一心一意谋发展，全面完成了“十五”计划，经济发展呈现出速度较快、效益较好、结构逐步改善、协调性逐渐增强的良好态势。

　　经济持续快速增长，质量和效益明显提高。全市生产总值年均增长１３．７％，比“九五”时期快５．２个百分点，２００５年生产总值达到２０７．９３亿元，是２０００年的２．５７倍。人均生产总值达到８４０美元。财政总收入年均增长２７．６％，２００５年达到２７．４亿元，较“九五”末增长２．４倍。

　　固定资产投入大量增加，重点项目建设顺利推进。累计完成固定资产投资３２６．３７亿元，是“九五”期间的２．８５倍。以交通为重点的基础设施建设取得重大进展，形成了西南地区重要的铁路交通枢纽，实现了市区到省城通高等级公路、县县通油路、乡乡通公路；以“西电东送”为重点的能源建设实现历史性突破，盘南电厂、野马寨电厂、发耳电厂、响水煤矿、发耳煤矿等一批重大项目相继开工建设。

　　经济结构调整步伐加快，新的经济发展格局正在形成。进一步加强“三农”工作，农业和农村经济稳步发展。农业结构调整力度加大，优势特色农业发展步伐加快，畜牧业产值占农业总产值的比重由２０００年的２７．１％升至２００５年的３６．１％。工业经济保持较快发展，工业增加值年均增长１７％。第三产业发展进一步加快。非公有制经济不断发展壮大，占生产总值的比重达２７．５％，比２０００年提高了５个百分点。

　　城市建设力度加大，城镇化水平进一步提高。市区基础设施建设力度加大，城市功能不断增强，城市形象明显改善。钟山大道、玉舍供水工程、岔河垃圾填埋场、市区防洪工程等一批重大项目建成并投入使用。着力打造“凉都”品牌，成功举办了两届“凉都”消夏文化节。红果、平寨及其他重点小城镇建设步伐加快。全市城镇化水平达到２６．２％。

　　社会事业全面进步，人民生活逐步改善。计划生育工作水平明显提高，２００５年人口自然增长率为７．６２‰、人口计生率为９５．８７％。教育事业加快发展，全市“两基”攻坚目标提前一年实现，组建了六盘水职业技术学院，实现了六盘水师范高等专科学校面向省内外招生和招收本科生的历史性突破，正式启动了六盘水矿业学院筹建工作。科技、文化、卫生等各项社会事业全面进步。城镇居民人均可支配收入达８０３２元，农民人均纯收入达１８６３元。五年间累计解决了２０万农村贫困人口的温饱问题。改革开放不断深入，民主法制建设、精神文明建设不断加强，民族团结、社会稳定的局面进一步巩固。

　　２００５年，全市经济保持了持续较快增长的良好势头，为全面完成“十五”计划画上了圆满句号。生产总值增长１７．６％；财政总收入和地方财政收入分别增长４２．７％和３６．４％；完成全社会固定资产投资１２１．４亿元，增长４１．３％；规模以上工业增加值增长１８．２％；城镇居民可支配收入和农民人均纯收入扣除物价因素实际分别增长１１．１％和７．５％；社会消费品零售总额增长１７．６％。

　　各位代表：“十五”期间我市经济社会发展取得的成绩，为我市“十一五”又快又好发展奠定了坚实基础。这些成绩的取得，是省委、省政府正确领导，省直各部门大力支持的结果；是全市各级各部门全面落实科学发展观的结果；是全市各族干部群众抢抓机遇、艰苦奋斗的结果。在此，我代表市人民政府，向在过去五年中为六盘水市经济社会发展作出艰苦努力和付出辛勤劳动的全市广大工人、农民、知识分子、干部，向各民主党派、工商联、无党派爱国人士和各人民团体，向驻市人民解放军、武警官兵、公安干警、民兵和预备役人员，向所有关心和支持我市改革开放和现代化建设的各界人士，表示衷心的感谢并致以崇高的敬意！

　　在肯定成绩的同时，我们也清醒地看到发展中存在的矛盾和问题，主要是：经济总量小，产业结构单一，粗放型经济增长方式尚未得到根本转变；农业生产条件差，农民收入增长缓慢，城乡二元结构突出；生态环境脆弱，加快发展与资源的合理开发和环境保护的矛盾加剧；社会事业发展相对滞后，劳动者素质亟待提高，就业和社会保障仍然面临很大压力；经济社会发展的体制性机制性约束不容忽视，社会利益关系处理难度加大，安全生产、社会治安等方面还存在许多薄弱环节，等等。对于这些矛盾和问题，我们要高度重视，采取有力措施加以解决。

“十一五”时期国民经济和社会发展的主要任务

　　“十一五”是我市加快小康社会建设、实现经济社会发展历史性跨越的关键时期。国家西部大开发的政策环境、全省“煤－电－化”发展战略的实施、我市“十五”奠定的坚实基础、全市广大干部群众思发展谋发展的愿望更加强烈，我市已经具备了加快发展的良好条件。我们一定要增强信心、振奋精神、努力拼搏、奋勇前进。

　　“十一五”时期我市经济社会发展的总体要求是：以邓小平理论和“三个代表”重要思想为指导，以科学发展观统领经济社会发展全局，解放思想，实事求是，与时俱进，能快则快，抢抓西部大开发和新阶段扶贫开发机遇，发挥资源、区位和大企业相对集中等优势，以改革开放和科技进步为动力，积极推进经济结构调整和经济增长方式转变，大力实施科教兴市、人才强市、生态立市、开放带动和可持续发展战略，以工业化带动城镇化，以城镇化推进工业化，着力发展煤炭、电力、钢铁、建材、煤化工、高载能等优势产业及其后续产业，努力在社会主义新农村建设、重化工基地建设、基础设施建设、城镇建设、生态环境建设、和谐社会建设等方面取得突破，开创社会主义经济建设、政治建设、文化建设、社会建设的新局面。

　　“十一五”时期我市经济社会发展的主要奋斗目标是：到２０１０年，全市生产总值比２０００年翻两番以上，达到５００亿元；人均生产总值比２０００年翻两番以上，达到１９００美元；单位生产总值能源消耗比“十五”期末降低２０％左右。基本建成重要的能源、原材料和煤化工基地；基本实现小康、步入社会主义新农村；基本建成和谐文明、现代开放的中心城市。

　　“十一五”时期我市经济社会发展的主要指标是：生产总值年均增长１５％以上；财政总收入年均增长１８％以上；社会消费品零售总额年均增长１３％以上；五年累计完成固定资产投资比“十五”期间增长１．８倍左右；城镇居民可支配收入和农民人均纯收入年均增长８％以上；到“十一五”期末，人口自然增长率控制在８‰以内；城镇登记失业率控制在４．２％以内。这些目标是预测性的，在具体执行过程中可根据实际情况适时适当调整。

　　一、加快社会主义新农村建设，扎实做好“三农”工作

　　按照“生产发展、生活宽裕、乡风文明、村容整洁、管理民主”的目标要求，遵循“规划先行、分类指导，群众自愿、政府支持，分步实施、逐步推进”的工作原则，加快社会主义新农村建设，扎实做好“三农”工作，使农村面貌有显著改善。

　　（一）全面发展农村经济，努力增加农民收入。充分挖掘粮食生产潜力，稳定提高粮食综合生产能力，到２０１０年粮食总产量达８５万吨左右。加强基本农田及其配套基础设施建设，保持耕地总量平衡，提高耕地质量，五年改造中低产田土２０万亩，新增、恢复和改善有效灌溉面积２０万亩，到２０１０年建成农业人口人均０．５亩高产稳产农田。大力推广优良品种和农业适用技术；在抓好粮食生产的同时，切实加快农业结构调整。稳定发展烤烟、油菜等传统经济作物，大力发展草食畜牧、蔬菜、马铃薯、茶叶、中草药和经果林等特色农业。坚持把加快畜牧业发展作为农业结构调整的突破口来抓，加快畜牧业繁育体系、疫病防治体系和饲草饲料体系建设，力争到２０１０年畜牧业产值占农业总产值的比重达４０％以上，肉类总产量达１８．５万吨以上。大力发展龙头企业，加强农产品基地建设，加快农业产业化经营；加快发展农村二、三产业，推动工农一体化、城乡一体化发展；在加强培训的基础上，引导和组织好劳务输出，大力发展劳务经济。力争每年组织劳务输出２万人以上。大力促进农村富余劳动力向非农产业转移、向城镇转移，五年累计转移农村人口３０万人左右。

　　（二）大力发展农村公共事业，着力提高农村人口素质。切实把提高农村人口素质作为发展农业和农村经济的重点来抓，全面发展农村教育、文化、卫生、计划生育等公共事业。建立和完善农村职业技术培训体系，加强对农民的素质培养和技能培训，加强农民思想道德建设，全面提高农村人口的整体素质。加大对村级干部、致富带头人以及农村医务人员、兽医人员等乡土人才的培养。加快发展农村文化事业。改善农村医疗卫生三级网络，大力发展新型农村合作医疗，力争到２０１０年全市新型农村合作医疗覆盖率达到８０％以上。完善农村计划生育家庭奖励扶助制度，大力实施“少生快富”工程。加强以村寨道路硬化、改房建池、改厕改圈、排水排污为主要内容的文明村寨建设，不断改善农村人居环境。到“十一五”末，累计解决３．６万户农村贫困户住房问题。与发展畜牧业相配套，加快农村沼气池建设，力争１／５的农户稳定用上沼气。

　　（三）扎实推进新阶段扶贫开发，努力改变农村贫困面貌。坚持把新阶段扶贫开发作为“三农”工作的重中之重，抓住改善基本生产生活条件、拓宽基本增收门路、提高基本素质三个关键环节，综合采取开发式扶贫、易地扶贫搬迁、长期救助等措施，因地制宜抓好整村推进、产业化扶贫、劳动力就业培训和转移等工作。“十一五”初期基本解决现有贫困人口温饱问题，“十一五”末基本实现低收入人口稳定脱贫。

　　（四）加快建立和完善农村服务体系，促进农村经济发展。加强农业技术推广、农产品质量安全和动植物病虫害防控体系建设，搞好农业生产资料供应和市场管理；加强信息服务体系和农产品市场体系建设，鼓励、引导和支持农民经纪人、农民专业协会、专业合作经济组织的发展；加强农村金融服务体系建设，规范发展适合农村特点的金融组织，大力推广农村小额信贷。

　　（五）深化农村各项改革，增强农村经济发展活力。坚持“多予、少取、放活”和工业反哺农业、城市支持农村的方针，稳定、完善和强化扶持农业的政策；稳定并完善以家庭承包经营为基础、统分结合的双层经营体制。认真贯彻执行涉农法律法规，依法保障农民的土地承包经营权及其他各项权利；巩固农村税费改革成果，全面完成以乡镇机构、农村义务教育和县乡财政体制等为主要内容的综合配套改革，切实化解乡村债务，建立减轻农民负担的长效机制。积极推进粮食流通体制、林业产权制度、供销社等方面的改革；加强村级组织建设，健全村民自治机制，大力推进村务公开，提高村级民主化管理水平。积极发展村级集体经济。

　　二、加快经济结构调整，大力发展优势特色产业

　　（一）大力发展工业经济。以基本形成具有较强竞争力的优势特色产业为目标，进一步依托境内大企业发展工业经济，积极引进优强企业，推进企业强强联合，拓宽投资领域，丰富产业门类，从单一结构向多元化发展；大力培育后续支柱产业，重点发展煤化工和高载能产业，加快推进煤炭资源综合开发，延长产业链、提高附加值；走新型工业化道路，淘汰落后生产能力和生产工艺，加强企业技术改造，大力节约能源资源，积极发展高新技术产业，从粗放型向精细化、集约化、循环式发展。“十一五”期间，要突出抓好以下三个方面：

　　第一，做大做强煤炭、电力、钢铁、建材等传统产业。在改造提高现有煤矿生产水平的同时，重点抓好盘南、发耳、黑塘、格目底、神仙坡、土地垭、盘东等矿区建设。积极整合煤炭资源，坚持走规模化、集约化和集团化的发展路子，到２０１０年建成２－３个年产原煤１０００万吨以上的大型企业集团，全市煤炭产量达到８０００万吨以上。继续实施“西电东送”工程，加快盘南电厂、发耳电厂建设，积极推进六枝电厂、野马寨电厂扩容、盘县电厂三期、盘东电厂等火电厂前期工作。基本建成万家口子、毛家河、鳝泥坡、泥猪河、响水电站二期等水电站，开工建设盘县坡上牧场风力发电站。力争到２０１０年电力装机容量达９００万千瓦以上。加强电网建设，确保城乡用电需要。大力支持水城钢铁（集团）公司整合资源、调整产品结构、提高技术装备水平、实施多元化发展，尽快建成年销售收入达１００亿元以上的大型钢铁联合企业。大力发展水泥、制砖等建材工业。对有条件的水泥企业进行兼并重组和技改扩能，扩大生产规模，力争水泥产量达３００万吨以上。引进新技术，以煤矸石、粉煤灰、钢渣等为主要原料，积极开发新型建筑材料。

　　第二，大力发展煤化工和高载能产业。按照“着力发展煤焦化、积极推进煤液化和煤气化、加快煤层气勘探和开发”的思路，大力发展煤化工产业，力争在“十一五”期间取得突破。按照“高起点、集约化、规模化”的原则，大力发展高载能产业，力争到２０１０年就地转化电能１５０亿度以上。重点抓好六盘水双牌铝业公司的技改扩能和铝材深加工项目，加快六枝煤、电、化、冶一体化项目的前期工作并开工建设。发展煤化工和高载能产业，对新上项目要严格把关，认真执行行业准入标准。综合采取经济、法律、行政等手段，限期淘汰改良焦、小机焦、小冶炼等落后生产能力。

　　第三，加快发展其他产业。围绕主导产业，大力发展矿山机械、机电产品及其他配套产业。结合农业结构调整和农业产业化的推进，加快发展轻工业，力争在畜产品、马铃薯、生姜、玉米等农产品加工上取得大的进展。加大对农业产业化龙头企业的扶持，力争培育一批产值上亿元的龙头企业。加快红山、石桥等轻工业集中区的规划和基础设施建设，大力引进轻工企业入驻。积极发展医药工业和日用消费品工业。积极培育电子信息、新型材料、生物技术等高新技术产业，力争取得较大进展。

　　（二）大力发展非公有制经济。认真贯彻落实《国务院关于鼓励和引导非公有制经济发展的若干意见》，清理和修订非公有制经济发展的政策，整顿和规范政府部门对非公有制经济的服务，切实为非公有制经济发展创造良好的政策环境、法制环境、舆论环境、服务环境。鼓励非公有制经济进入农业、工业、服务业、公用事业、基础设施等领域，鼓励非公有制经济与其他所有制经济联合、参股，加快发展混合所有制经济。引导金融机构提高对非公有制企业的贷款比重，规范发展中小企业信用担保体系，进一步支持非公有制经济加快发展；引导非公有制经济调整组织结构，强化内部管理，提高企业素质，增强发展活力。到“十一五”末，非公有制经济的发展质量和效益明显提高。

　　（三）大力发展服务业。积极运用现代经营方式和服务技术改造提升传统服务业，着重发展商贸流通、交通运输、市政服务等行业。特别是随着交通条件的不断改善，要加快发展城乡客货运输业。加强现代物流基础设施和专业市场建设，积极培育现代物流业。大力发展并规范信息、法律、咨询、会计、公证、经纪、代理等现代服务业。面向城乡居民消费，进一步促进房地产业健康发展；围绕“凉都”品牌的打造和“乌蒙山国家地质公园”基础设施的建设，开发和保护旅游资源，积极培育旅游产业；加快发展社区服务业，提供便民利民服务；引导文化娱乐、教育培训、体育健身、卫生保健等产业发展，满足服务性消费需求；发展农村服务业，增加服务内容，改善消费环境。力争“十一五”期间服务业增加值年均增长１３％以上。

　　三、努力扩大固定资产投资，保持对经济增长的强劲拉动

　　（一）大力争取国家和省的投入。抢抓国家设立西部开发专项资金的机遇，加大项目前期工作力度，高质量地准备基础设施、优势产业和关系群众切身利益的公益性项目，积极争取国家、省在能源、优势原材料、旅游等领域率先进行产业投资基金试点，力争有更多的项目列入国家、省建设规划。在继续争取国家、省增加对我市一般性转移支付的同时，以石漠化治理、采煤沉陷区治理等工程为重点，争取国家、省加大对我市的专项投入力度。

　　（二）强化企业融资。以大企业为依托，以优势资源和大项目为载体，以资本为纽带，打破地域、行业和所有制等界线，采取重组、兼并、参股等方式，推进企业间强强联合，以投资主体多元化和产权多元化拓宽融资渠道。支持具备条件的企业发行企业债券、上市融资。

　　（三）努力激活民间资金。要在进一步加大招商引资工作力度，大力吸引境外资金参与我市经济建设的同时，把激活民间资金作为扩大固定资产投资的重点来抓。放宽投资准入限制，利用特许经营、投资补助、贷款贴息、税收优惠等多种措施，鼓励、支持和引导社会资本以独资、合资、合作、联营、项目融资等方式，参与经营性的公益事业和基础设施项目建设。通过水价、收费公路收费标准的合理调整、建立生态建设和环境保护补偿机制等措施，鼓励社会资本参与市政、公路、生态等项目建设。

　　（四）切实规范政府投资行为。积极调整财政支出结构，重点投向市场不能配置的公共设施和公益性项目建设。充分发挥政府投资的启动和导向作用。积极稳妥地推进供水、供气、排污、垃圾处理等市政公用事业改革。结合国有资产管理体制改革，按照所有权与经营权分离的原则，整合和盘活国有经营性资产。切实抓好城市土地的清理、收购、储备、开发和招标拍卖、挂牌出让工作，收好、管好、用好煤炭价格调节基金、运煤公路维改费、城市基础设施建设配套费，增强政府的投资能力和偿债能力。

　　四、加大基础设施建设力度，进一步夯实经济发展基础

　　（一）加快交通基础设施建设。以基本建立起适应经济社会发展需要的综合交通运输体系为目标，大力加强铁路、公路等交通基础设施建设。积极争取上级支持，做好协调服务，建成六盘水至沾益复线，并以六盘水客站换边、新建发戛坡货场为重点，配套完善六盘水铁路枢纽；围绕重化工基地发展，改建或新建一批专用铁路支线；力争开工建设南宁至昆明铁路复线；积极开展滇黔二线铁路等项目的前期工作。按“四个层次”整体推进公路建设，实现县县通高速公路或高等级公路、乡乡通油路、村村通公路。建成镇宁至胜境关高速公路境内段，开工建设杭瑞高速公路和六盘水至兴义高速公路境内段；建成红果至威舍、柏果至火铺、马场桥至玉舍、英武至柏果、平寨经郎岱至毛口等二级公路，力争开工建设大河经连山经南开至青林、大山哨经六枝经水城至威宁、水城至赫章、米箩至羊场、英武至大山等二级公路。基本完善乡镇汽车站（点）建设。强化公路管养，确保安全畅通。加快六盘水支线机场的前期工作，力争开工建设。

　　（二）加快水利基础设施建设。坚持“大中小微相结合、以中小水利设施为主，蓄引提排节相结合、以蓄节水为主”，以解决工程性缺水为重点，统筹抓好城镇供水、农村人畜饮水、工业用水、农田灌溉、防洪排涝等水利工程建设。加强市区和平寨、红果两个县城的供水能力建设，配合抓好黔中水利枢纽工程，开工建设双桥、白河沟、岱瓮等供水工程。加大力度实施农村饮水安全工程，抓好一批小微型水库和小水窖建设，到２０１０年基本解决小城镇和农村人畜饮水问题。围绕工业项目布局，加快配套水利工程建设，保证工业用水需要。与基本农田建设相配套，开工建设发耳片区、木岗片区等农田灌溉工程，完成病险水库治理，大力实施“益民工程”和“烟水配套工程”。建设一批城镇和农田防洪排涝工程。加强水资源的统一调度和管理，控制开采地下水，推进节水型社会建设。到２０１０年，初步实现全市城乡经济社会发展供水安全、饮水安全、防洪安全和水生态环境安全的目标。

　　（三）加快信息基础设施建设。完成市区至县、特区的长途光缆干线建设和有线网络的数字化改造，扩大城乡有线和无线通信覆盖率。坚持资源共建共享原则，积极推进电信网、有线电视网和互联网的三网融合。继续推进电子政务建设，加快公共服务综合信息平台建设。加快建立电信普遍服务机制和邮政普遍服务补偿机制，支持普及农村电信和邮政服务。

　　五、加快推进城镇化，增强对经济发展的辐射和带动

　　坚持以工业化带动城镇化，按照新区开发、旧城改造、小城镇建设、新农村建设“四轮驱动”的要求，加快推进城镇化进程。以城镇规划为依据、以制度创新为动力、以功能培育为基础、以加强管理为保证，按照循序渐进、节约土地、集约发展、合理布局的原则，完善相关配套政策，进一步加快市区和红果、平寨两个县城的发展，继续抓好重点小城镇建设，力争到２０１０年全市城镇化水平达到３５％以上。

　　按照“绿化、美化、文化、现代化”的要求，瞄准把市区建设成为“设施配套，功能完善；要素聚集，产业发展；景致优美，环境怡人；文明诚信，和谐有序”的现代化城市的目标，切实搞好城市规划、建设和管理。完成市区总体规划第三轮修编。加快市区基础设施建设，完善城市功能。高标准、高质量建设城市新区。大力开展小街小巷治理，加快旧城和“城中村”改造。抓好环城绿化带和各种公共设施建设，着力改善人居环境。切实克服重建轻管现象，改进城市管理方式，促进城市管理上台阶。加强社区建设，完善社区功能。大力开展全国文明卫生城市创建活动。树立城市经营理念，大力发展城市经济。同时，切实加强对红果、平寨的规划、建设和管理。

　　要把城镇化发展的重点放在部分基础条件好、发展潜力大的乡镇，使之尽快完善功能，集聚人口，发挥农村地域性经济、文化中心的作用。要把繁荣小城镇经济放在重要位置，把引导乡镇企业合理集聚、完善农村市场体系、发展农业产业化经营和社会化服务与小城镇建设结合起来，加快推进一批各具特色的重点小城镇建设。深化土地、户籍和公共服务等方面的改革，提高城镇的综合承载能力。

　　六、大力实施可持续发展战略，切实转变经济增长方式

　　（一）加强人口与计划生育工作。坚持控制人口数量和提高人口素质并重。建立健全计划生育利益导向机制。综合治理出生人口性别比例失调，实施出生缺陷干预工程，提高出生人口素质，改善人口结构。加强流动人口计划生育管理与服务。加强基层计生服务网络建设，提高技术装备水平和服务能力。到２０１０年，人口出生率控制在１４‰以内，进入并稳定低生育水平。

　　（二）合理开发和保护资源。坚持开发与节约并重、节约优先，力争到２０１０年建设资源节约型社会取得实质性进展。加快做好土地利用总体规划修编。实行最严格的耕地保护制度，严格执行耕地占补平衡制度，严格控制非农建设用地占用耕地特别是基本农田，鼓励建设项目多用坡地、闲置地和非耕地，节约和集约利用土地。依法保护和开发水、能源、矿产等重要资源。大力整顿和规范矿业秩序。加强对矿山开采回采率、采矿贫化率和选矿回收率的监督管理，确保矿产资源综合回收率在“十五”末的基础上提高５个百分点以上，煤炭回采率达到５０％以上。

　　（三）加强生态建设和环境保护。生态建设方面，要以基本建成“两江”上游重要生态屏障为目标，继续加大以退耕还林工程、天然林保护工程、水土流失综合治理和石漠化综合防治为重点的生态建设力度。特别要抓好退耕还林后续产业的培育，切实解决退耕还林农户当前和长远生计，巩固退耕还林成果。着力解决农村能源问题。力争“十一五”期间全市森林覆盖率每年增加１个百分点以上，到２０１０年达到３５％左右。环境保护方面，一是要把好源头关。对新上项目要严格执行环境影响评价法和“三同时”制度；二是要把好生产关。大力推进清洁生产，严格执行排污总量控制、排污许可，减少污染物排放；三是要把好治理关。加大对重点区域、重点企业、重点污染物的综合整治，实现城镇周围、主要河流沿线和主要公路沿线工业企业污染物排放全面达标。加大矿山生态恢复治理力度，积极预防和治理采煤沉陷区的地质灾害。

　　（四）大力发展循环经济。把大力发展循环经济作为推进经济增长方式转变的重点，贯穿到资源开发和产业发展的各个领域，力争到“十一五”末建设循环经济型社会取得积极进展。工业方面，要在做好规划的基础上，争取把我市列为全省重化工循环经济试点城市，并选择一些企业开展试点。加大对工业固体废弃物的利用力度。同时，结合发展煤化工和高载能产业，积极推进生态工业园区建设。农业方面，抓好生态农业示范基地建设，大力发展农业和农村循环经济。其他行业也要按照发展循环经济的要求，积极探索循环经济发展模式。加大对发展循环经济和建设节约型社会的宣传，努力形成全社会的共同行动。

　　七、大力实施科教兴市和人才强市战略

　　（一）坚持优先发展教育事业。大力实施教育优先发展战略，基本建立起适应经济社会发展的国民教育体系和人才培训体系。重点抓好四个方面：一是要全面巩固和提高“两基”攻坚成果，确保适龄儿童入学率达到９９％以上。认真落实“两免一补”政策，实行真正意义的义务教育。二是要大力发展高中阶段教育，力争到２０１０年高中阶段毛入学率达到５０％左右。三是要加强职业教育。认真贯彻落实《国务院关于大力发展职业教育的决定》，加快建立职业教育和培训网络。重点发展中等职业教育，力争到２０１０年中等职业学校在校生达３万人左右。加快发展高等职业教育，完善六盘水职业技术学院。四是要积极发展高等教育。建立六盘水矿业学院，在六盘水师范高等专科学校的基础上申办六盘水学院。同时，要大力促进电视教育、远程教育、民族教育、民办教育、幼儿教育等各类教育协调发展。继续增加对教育的投入，加强教育基础设施建设。加强对教师尤其是农村教师的培训，全面实施素质教育。大力整合教育资源，实施教育均衡发展，努力促进教育公平。积极探索多种所有制的办学模式，吸引外来资金、民间资金投入教育事业。鼓励和支持民办教育加快发展。

　　（二）加快科学技术发展。紧紧围绕经济社会发展，大力推进科技进步和技术创新。充分发挥政府在科技创新中的主导作用、市场在科技资源配置中的基础作用、企业在科技创新中的主体作用，形成科技创新的整体合力。多渠道筹措科技研发经费，力争到“十一五”末全社会科技研发投入占生产总值的比重达到１％左右。依托高等院校、科研单位和企业科技中心，加大科技成果的引进和转化力度。大力提高原始创新、集成创新特别是引进消化吸收再创新的能力。支持民营科技企业加快发展。加大农业科技示范园区建设力度。大力开展科普宣传，提高全民科技素质。

　　（三） 加强人才资源开发和利用。牢固树立人才是第一资源的观念，坚持党管人才的原则，全面加强党政人才、企业经营管理人才、专业技术人才三支队伍建设，促进总量增长、结构改善和素质提高。切实抓好培养、吸引、使用三个环节。推进农村乡土人才开发。进一步完善人才市场体系。建立科学的人才评价、选拔任用和激励保障机制，为各类人才成长和发挥作用营造良好的环境，在全社会形成重才、爱才、聚才、用才的良好氛围。

　　八、进一步深化改革扩大开放，不断增强经济发展的活力

　　（一）大力推进国有企业改革。以促进企业发展为目的，以产权制度改革为重点，全面完成地方国有企业改革改制，完善法人治理结构，全面建立现代企业制度。稳步推进国有困难企业依法关闭破产，完成国有商贸流通、交通和建筑等企业改革，促进国有资本退出一般性竞争领域。积极发展混合所有制经济，努力探索公有制的多种有效实现形式。完善国有资产监管体制，完善国有企业负责人经营业绩考核体系和激励约束机制，实现国有资产保值增值。

　　（二）进一步深化投资体制、财政体制和其他各项改革。认真贯彻落实《国务院关于投资体制改革的决定》，逐步建立新型投资体制。进一步加强投融资主体建设，落实企业投资主体地位，规范政府投资行为。完善项目审批、核准和备案的相关规定，完善项目审批方式，进一步简化行政审批程序。按照建立公共财政的要求，合理调整财政支出结构，进一步完善财政管理体制。继续深化部门预算、国库集中支付、政府采购和收支两条线管理制度改革，积极推进“乡财县管乡用”财政管理方式改革。支持金融系统加快改革，加快城市商业银行建设，稳步发展农村金融，发挥金融对地方经济的支持作用。大力加强金融生态环境建设。深化交通、水利等管理体制改革，积极推进教育、科技、文化、卫生、体育、广播电视等各项社会事业改革。积极推进现代市场体系建设。继续整顿和规范市场经济秩序。

　　（三）努力扩大对外开放。加强投资环境特别是软环境建设，进一步完善招商引资工作协调机制、服务机制和投诉处理机制。进一步拓宽开放领域。改进招商方式，提高引资实效。积极发展进出口贸易。切实加强与友好城市和对口帮扶城市的经济文化交往，积极参与“泛珠三角”等区域经济、技术、信息的合作与交流。加强与周边地区的协作。

　　九、切实解决群众的切身利益问题，努力构建和谐社会

　　（一）切实做好就业再就业工作。认真贯彻落实《国务院关于进一步加强就业再就业工作的通知》，围绕经济发展多渠道开发就业岗位，努力扩大就业；推进劳务协作，搞好劳务输出，积极转移就业；改进就业服务，强化职业技能培训，加强劳动力市场建设，切实促进就业；开展失业调控和就业管理，加强劳动仲裁和劳动执法监察，规范企业裁员，规范企业用工行为，全面推进职业技能鉴定工作，实行职业资格证书制度，确保规范就业；努力做好城镇新增劳动力、大中专毕业生、复员退伍军人、农村富余劳动力和失地农民的就业工作，做到统筹就业。“十一五”期间，力争每年促进城镇就业２万人以上。

　　（二）加快完善社会保障体系。要以基本建立城乡保障体系、健全社会保障分担机制、提高保障水平为目标，着力完善“三大体系”：一是完善最低生活保障体系。继续做好城镇居民最低生活保障工作，实现“应保尽保”。逐步建立农村居民最低生活保障制度。二是完善社会保险体系。继续完善城镇职工基本养老、基本医疗和失业、工伤、生育等保险制度，努力扩大参保人口覆盖面。到“十一五”末，城镇失业、养老、医疗保险覆盖率达到６０％以上。多渠道筹措社会保险基金，逐步做实养老保险个人帐户，改革基本养老金计发办法。积极发展农村社会保险、农村养老保险、农村医疗保险等，努力扩大社会保障覆盖范围。把失地农民和进城务工人员作为发展社会保障的重点，逐步纳入社会保障覆盖范围。三是完善社会救助体系。进一步做好城镇生活无着和流浪乞讨人员的救助工作。认真落实农村“五保”供养政策。切实把农村特困群众全部纳入定期救助范围。支持社会慈善、社会捐赠、社会福利、群众互助等社会扶助活动。切实做好救灾救济工作。

　　（三） 着力提高人民群众的健康素质。卫生方面，一是要加强城乡公共卫生设施建设，重点抓好乡镇卫生院和村级卫生室建设；二是要加强卫生队伍建设，着力提高医务人员素质，特别要加强对乡村医务人员的培养和培训；三是要加快医疗卫生体制改革，强化卫生执法监督，规范医疗市场，整顿药品流通秩序，降低医疗费用。同时，要着力抓好艾滋病、地氟病等重点传染病、地方病综合防治工作，建立健全突发公共卫生事件应急处理等长效公共卫生机制。体育方面，要大力发展群众体育，有重点地发展竞技体育。加快体育设施建设，建成凉都体育中心。

　　（四） 丰富人民群众精神文化生活。加快市“五馆”、县“两馆”和乡镇文化服务中心等文化基础设施建设。深化文化体制改革，大力发展文化产业。加强文化市场建设，依法规范文化市场秩序，形成健康文明向上的市场环境。加大民族民间文化的发掘、整理和保护力度。抓好有线广播电视网络整合和产业化改制，加快广播电视数字化和农村通讯设施建设，实现农村广播、电视、电话村村通，乡（镇）有线广播电视网络覆盖率达８０％以上，广播、电视人口覆盖率分别达９５％和９８％以上。

　　（五） 切实保障人民群众的生命财产安全。正确处理好安全与生产的关系，按照“安全第一、预防为主、综合治理”的方针，切实加强安全生产工作，确保安全生产状况进一步好转，力争到“十一五”末亿元生产总值死亡人数下降到１人以内、煤炭百万吨死亡人数下降到３人以内、万车死亡人数下降到１０人以内。坚持以煤矿、道路交通和消防为重点，加大安全设施建设力度，不断改善安全生产条件。健全安全生产监管体系，强化安全执法检查，严格执行有关法律法规和规章制度。强化企业的责任主体和投入主体地位，完善和落实安全生产责任制和安全事故责任追究制。加大自然灾害预测预报和防灾减灾工作力度。加强对药品、食品、餐饮卫生的市场监管，保障群众健康安全。

　　（六） 切实加强社会建设和管理。健全党委领导、政府负责、社会协同、公众参与的社会管理格局。切实加强对各种行业协会和同业公会的教育、管理和引导。加强和谐社区、和谐村镇建设。合理调节收入分配，努力维护社会公正。正确处理新形势下的人民内部矛盾，认真做好人民群众来信来访工作。建立健全社会预警体系和应急救援、社会动员机制，提高处置突发事件能力。大力加强社会治安防控体系建设，落实综合治理的各项措施，深入开展“平安六盘水”创建活动，打好禁毒人民战争，切实维护社会稳定。

　　十、大力加强社会主义精神文明和民主法制建设

　　（一）大力加强社会主义精神文明建设。坚持以马列主义、毛泽东思想、邓小平理论和“三个代表”重要思想为指导，全面贯彻落实科学发展观，以构建和谐社会为目标，坚持解放思想、实事求是、与时俱进。努力提高人民群众的科学文化素质，引导广大干部和群众树立正确的世界观、人生观、价值观，切实遵守社会公德、职业道德、家庭美德。广泛开展“热爱六盘水、建设六盘水”主题教育活动，在全社会倡导爱国守法、明礼诚信、团结友善、勤俭自强、敬业奉献的基本道德规范。继续广泛深入地进行党的基本路线和基本纲领教育，进行爱国主义、集体主义、社会主义和艰苦创业精神教育。加强农村精神文明活动阵地建设。继续广泛开展各种形式的群众性精神文明创建活动。

　　（二）大力加强社会主义民主政治和法制建设。各级政府要自觉接受同级人大及其常委会的法律监督和工作监督，自觉接受同级政协的民主监督，认真听取民主党派、工商联、无党派人士和各人民团体的意见，接受新闻舆论和社会公众监督，重视人民群众对政府机关及其工作人员的监督，支持监察、审计部门依法独立履行监督职责。全面贯彻党的民族政策，巩固和发展平等、团结、互助、和谐的社会主义民族关系。加快民族地区经济发展。扩大基层民主，加强基层自治组织建设，坚持和完善职工代表大会和其他形式的企事业民主管理制度。发挥工会、共青团、妇联等人民团体广泛联系群众的桥梁纽带作用。大力支持国防后备力量和武装警察部队建设，增强全民国防意识，提高国防动员能力，做好民兵预备役工作，加强“双拥”工作，巩固和发展军政军民团结。进一步做好宗教、侨务和对台工作。全面实施妇女、儿童发展规划，切实维护妇女、儿童合法权益。积极发展老龄事业和残疾人事业。大力推进法制建设，切实加强法律援助和基层法律服务工作，继续做好普法依法治理工作，努力提高全民法律素质。

加强政府自身建设

　　切实加强政府自身建设，是完成“十一五”规划的重要保障。我们要按照“务实、高效、公正、廉洁”的要求，抓好以下四个方面的工作：

　　一、全面正确履行政府职能

　　深化政府行政管理体制改革，进一步加快政府职能转变，切实将政府职能转到经济调节、市场监管、社会管理和公共服务上来，使政府管理经济的方式从直接管理为主转变为间接管理为主，从运用行政手段为主转变为运用经济和法律手段为主，从前置审批为主转变为后续监管为主。更加注重履行社会管理和公共服务职能，把更多的工作精力和公共资源投入到促进社会事业发展和解决民生问题上来。处理好政府、市场、企业三者的关系，明确规范政府职能定位，加快推进政企分开、政资分开、政事分开、政府与市场中介组织分开，把政府该管的事情管好，把不该管的事交给市场、企业、社会组织和中介机构。

　　二、建设法治政府

　　认真贯彻落实国务院《全面推进依法行政实施纲要》，切实加强行政执法和行政监督。严格规范性文件的制定程序和备案审查，建立规章、规范性文件的定期清理制度。严格按照法定权限和程序行使权力、履行职责，做到有法必依、执法必严、违法必究；推进相对集中行政处罚权和综合执法，解决行政执法职责交叉、多头执法等问题。清理行政执法主体，健全行政执法依据、程序公开制度，严格执行行政执法责任制和过错责任追究制，完善行政执法监督制约机制。进一步健全科学民主决策机制，建立和完善重大问题集体决策制度、专家咨询制度、社会听证制度。

　　三、建设服务型政府

　　坚持立党为公、执政为民，坚持把维护好、实现好、发展好广大人民群众的根本利益作为政府工作的出发点和落脚点。从人民群众普遍关心和涉及人民群众切身利益的问题入手，大力推进政务公开，充分尊重人民群众的知情权、参与权和监督权，增强政府工作的透明度。坚持公道正派、大公无私，带着深厚的感情为群众服务。大力弘扬求真务实、真抓实干的精神，深入基层、深入实际，广泛开展调查研究，切实解决实际问题，力戒形式主义和官僚主义。切实提高工作效率，努力形成雷厉风行、一抓到底的工作作风。加强对重点工作、重大项目的跟踪监督，进一步健全责任制度、督查制度、考核制度和报告制度，确保各项工作落到实处。加强“诚信政府”建设，提高政府公信力。认真贯彻实施《公务员法》，加强公务员队伍建设，努力提高各级政府的服务水平和领导发展的能力。

　　四、建设廉洁政府

　　全面贯彻落实中央《建立健全教育、制度、监督并重的惩治和预防腐败体系实施纲要》，坚持标本兼治、综合治理、惩防并举、注重预防的方针，教育和督促政府系统的各级领导干部加强党性修养，牢记“两个务必”，自觉做到“八个坚持、八个反对”，常修为政之德、常思贪欲之害、常怀律己之心，筑牢拒腐防变的思想道德防线。切实加大查办违纪违法案件的力度，坚定不移地惩治腐败，加大抓源头治本的工作力度，为经济社会发展营造一个风清气正的环境。

　　各位代表：２００６年是实施“十一五”规划的开局之年。今年全市经济社会发展的主要预期目标是：生产总值增长１５％以上；全社会固定资产投资增长１０％以上；财政总收入增长１８％以上；社会消费品零售总额增长１５％以上；城镇居民人均可支配收入增长８％以上，农民人均纯收入增长８％左右；人口自然增长率控制在９‰以内；城镇登记失业率控制在４．３％以内；单位生产总值能耗降低４％以上。围绕上述目标，我们将抓好以下工作的落实：一是切实做好“三农”工作，稳步推进新农村建设。抓好粮食生产，力争粮食总产量增长２％以上。加快农业结构调整，大力发展畜牧业，力争畜牧业产值占农业总产值的比重提高１．５个百分点左右。加快培育新型农民，完成职业技能培训１万人，培训２５０名村级卫生员和２５０名村级兽医。大力引导农村富余劳动力向城镇和非农产业转移，有组织劳务输出４３００人以上。加强农业基础设施建设，解决６０００户农村贫困户的住房问题，解决３万农村人口的饮水安全问题。扎实做好扶贫开发工作，解决２．７５万贫困人口的温饱问题，使４．５万低收入人口稳定脱贫。二是加快工业结构调整，促进资源就地转化，推动产业结构优化升级。进一步加快大型煤矿建设和小型煤矿整合；力争煤化工发展在年内实现重大突破；大力发展高载能产业。继续做好经济运行调节工作，采取有力措施确保电煤供应。三是加强以交通和水利为重点的基础设施建设，继续扩大固定资产投资。四是加快城市建设，进一步完善城市功能。基本完成人民路改造；完成市区排污管网配套工程建设；完善市区小街道基础设施和主干道人行天桥。五是大力节约资源能源，加快建设资源节约型、环境友好型社会。加强重点行业和年耗能万吨标准煤以上企业的能耗、物耗管理。认真做好禁止生产和使用烧结实心砖的工作。六是加快以教育为重点的社会事业进步，努力促进经济社会协调发展。巩固和提高“两基”攻坚成果。 抓好六盘水矿业学院的筹建工作，确保年内招生。新建１所高级实验中学。抓好盘县、六枝特区和水城县新型农村合作医疗制度试点。开工建设凉都体育中心。七是大力推进改革开放，不断增强发展活力。加快现代企业制度建设。制定出台鼓励支持引导非公有制经济发展的具体政策。继续推进行政管理、投资、财税、金融等各项改革。大力改善投资环境，进一步扩大招商引资规模。八是认真解决群众的切身利益问题，积极构建和谐社会。继续做好就业再就业工作，逐步完善社会保障体系。切实加强安全生产工作，坚决遏制重特大事故的发生，把安全事故控制在省下达的指标范围内。加强社会治安综合治理，维护社会稳定。切实办好“十件实事”。

　　各位代表：未来五年是我市实现经济社会发展历史性跨越的关键时期。六盘水的发展充满希望，前景十分美好。让我们紧密团结在以胡锦涛同志为总书记的党中央周围，高举邓小平理论和“三个代表”重要思想伟大旗帜，按照科学发展观和构建社会主义和谐社会的要求，全面贯彻落实党的十六大、十六届五中全会和省委九届八次全会、市委四届八次全会精神，在省委、省政府和市委的领导下，团结和依靠全市各族人民，抓住机遇，用好机遇，扎实苦干，奋发图强，为全面完成我市国民经济和社会发展第十一个五年规划而努力奋斗！
